# Association of Health and Psychological Factors with Academic Achievement and Non-Verbal Intelligence in University Students with Low Academic Performance: The Influence of Sex

**DOI:** 10.3390/ijerph19084804

**Published:** 2022-04-15

**Authors:** Aniel Jessica Leticia Brambila-Tapia, Aris Judit Miranda-Lavastida, Nancy Araceli Vázquez-Sánchez, Nancy Lizbeth Franco-López, Martha Catalina Pérez-González, Gonzalo Nava-Bustos, Francisco José Gutiérrez-Rodríguez, Francisco Fabián Mora-Moreno

**Affiliations:** 1 Departamento de Psicología Básica, Centro Universitario de Ciencias de la Salud (CUCS), Universidad de Guadalajara, Guadalajara 44100, Mexico; gonzalonava2004@yahoo.com.mx (G.N.-B.); gutierrfrancisco@gmail.com (F.J.G.-R.); 2 Centro de Estudios sobre Aprendizaje y Desarrollo (CEAD), Departamento de Psicología Básica, Centro Universitario de Ciencias de la Salud (CUCS), Universidad de Guadalajara, Guadalajara 44100, Mexico; aris.miranda@academicos.udg.mx (A.J.M.-L.); nancy.vazquez@alumnos.udg.mx (N.A.V.-S.); nancylizbeth.franco@alumnos.udg.mx (N.L.F.-L.); 3 Centro de Evaluación e Investigación en Psicología (CEIP), Departamento de Psicología Básica, Centro Universitario de Ciencias de la Salud (CUCS), Universidad de Guadalajara, Guadalajara 44100, Mexico; catalina.perez@academicos.udg.mx

**Keywords:** academic achievement, non-verbal intelligence, psychological factors, admission exam test

## Abstract

Academic achievement, measured with the grade point average (GPA), is a stable characteristic that has been associated with many sociodemographic and psychological variables; however, the relation of these variables with GPA has not been totally elucidated. The objective of this study was to perform an association of health, psychological and personal variables with GPA and non-verbal intelligence in low-academic performance population according to sex. We invited health sciences university students who had failed the same subject twice to complete a set of sociodemographic and psychological variables and a non-verbal intelligence test. The GPA, admission exam test and preparatory GPA were obtained. We included 124 students, and found that GPA was associated with non-verbal intelligence in women but not in men; in whom, having a job and having a romantic partner, were more correlated. In women, positive relations with others, emotion perception and weekly physical activity hours were marginally correlated with GPA; while in men, emotion regulation and self-motivation had a tendency of correlation with GPA. In addition, we found that non-verbal intelligence was associated somatization and the number of diseases in women. Academic achievement is regulated by different variables in each sex; therefore, intervention programs addressed by sex are needed to increase it.

## 1. Introduction

Academic achievement, usually measured with the grade point average (GPA), is a very stable and heritable characteristic that remains constant over time [1]; however, it has been associated with a variety of sociodemographic, psychological and intellectual factors including positive associations with non-verbal intelligence, academic motivation, academic self-efficacy, emotional intelligence, task-oriented coping strategies, physical activity, sleep-related factors, conscientiousness and female sex, and negative associations with stressful life events, alcohol use, depression, stress, delinquent activity and avoidance coping [2,3,4,5,6,7,8,9]. In addition, intelligence has shown negative correlations with psychosocial adversities and low income, and positive correlations with maternal education [10]; as well as with specific personality traits [11]. To date, the socio-ecological outcomes model was the only found theoretical model related with academic achievement [12]; this suggests that interactions, among many factors including societal, environmental, intrapersonal and campus-based factors can influence the student success outcomes. In line with this model, a meta-analysis that investigated the influence of psychosocial factors in college students’ success, identified that some psychological variables, including motivation, self-perception, attribution and self-regulation contributed with a small but significant effect in academic achievement of college students [13] 

However, few studies have measured a comprehensive number of these variables, including non-verbal intelligence, health, psychological and personal ones, in order to associate them with GPA and non-verbal intelligence, and, to the best of our knowledge, no study has performed these associations in students with low academic performance. Therefore, the objective of this study is to identify the variables most associated, with bivariate and multivariate analyses, to GPA and non-verbal intelligence according to sex in a subgroup of health sciences university students with low academic performance, in order to detect which variables could be modified to increase academic achievement in this population. 

Therefore, as an initial hypothesis, we suggest that non-verbal intelligence and previous academic variables (including the admission exam test and preparatory GPA) are the most associated ones with current GPA; however, we also suggest that personal variables (mainly having a job, having children, maternal and parental schooling) are negatively and positively related with GPA (for the 2 first and the 2 last, respectively); that specific behavioral variables (including exercise, sleep quality, smoking and alcoholism), are positively and negatively associated with GPA, (for the 2 first and the 2 last, respectively), and finally, we hypothesize that specific psychological variables (mainly self-motivation and conscientiousness) are positively related with GPA. Finally, the inclusion of the health variables (somatization and the number of diseases) was performed in order to detect a possible association with GPA and non-verbal intelligence. 

## 2. Participants and Methods

Study population: Students of the health sciences university center and who had failed the same subject twice (these students represent around 1% of the university center population by semester and therefore, are considered with “low academic performance” by the research team) were invited to participate. Those who accepted, signed an informed consent and filled out a questionnaire with sociodemographic, behavioral and psychological variables, and afterwards, they performed a non-verbal intelligence test. All the information obtained was kept/held as confidential and used only for this research. 

Sociodemographic, health and behavioral variables: Age, sex, schooling, maternal and paternal schooling, number of siblings, whether they have a romantic partner, whether they have a job, whether they have children, number of daily study hours, monthly extra money, excluding necessary expenses (5 ordered ranges of extra money), number of daily free hours, number of daily recreative hours, weekly hours of physical activity, frequency of smoking, alcoholism and 6 types of drug consumption (marijuana, hashish, ecstasy, cocaine/crack, heroin, amphetamines): these frequencies were measured with 6 categories: never, 2–4 times a year, once in the month, many times in the month, once a week and many times in the week; the presence of 16 different diseases (diabetes, hypertension, overweight, thyroid problems, allergies, asthma, gastritis/gastric ulcer, colitis/irritable colon, migraine, acne, neurodermatitis, sinusitis, kidney/bladder problems, anorexia/bulimia, anxiety and depression problems that require medication) and any additional ones; somatization was measured with the Patient Health Questionnaire 15 (PHQ-15) [14]; self-reported weight and height to obtain the body max index (BMI), sleep satisfaction and sleep quality which were measured with the first item and the next 3 items (7 items in total because item 2 consists of 5 items) of the OVIEDO sleep questionnaire, respectively [15], and finally, we measured the quality of food intake with the Mini-ECCA scale [16]. 

Psychological variables: We measured academic stress with the academic stressor subscale of the SISCO scale [17], which was validated in Chilean student population [18]; depression with the Patient Health Questionnaire 9 (PHQ-9) [19]; anxiety with the Generalized Anxiety Disorder test (GAD-7) [20]; positive and negative emotions with the positivity-self scale (PSS) [21]; the 6 subscales of the shortened version of psychological well-being (PWB) scale (self-acceptance, autonomy, environmental mastery, personal growth, positive relations with others and purpose in life) [22]; optimism with the Life Orientation Test (LOT-R) [23]; personality with the reduced version of the NEO-FFI scale, which includes 5 subscales: neuroticism, openness, agreeableness, conscientiousness and extraversion [24], and finally, we measured 5–6 items of 3 subscales of the Trait Emotional Intelligence Questionnaire (TEIQUE): self-motivation (5 items), emotion perception (5 items) and emotion regulation (6 items) (these items are described in Supplementary File S1) [25].

Academic variables and non-verbal intelligence: The GPAs and the university entrance score (UES) were obtained from the university database. The UES was composed by the sum of the preparatory grades (preparatory GPA) and the admission exam test (college board exam) which includes: math and verbal reasoning and indirect redaction. Finally, non-verbal intelligence was measured with the Beta-4 test, which measures non-verbal intelligence (fluid and spatial non-verbal intelligence) and includes: coding, picture completion, clerical checking, picture absurdities and matrix reasoning [26]. 

### Statistical Analysis

Descriptive data were presented with means and standard deviations for continuous variables and frequencies and percentages for binary/categorical ones. Continuous variables were associated with Pearson and Spearman tests for parametric and non-parametric distribution, respectively, while *t*-test for independent samples was used to compare continuous variables between 2 different categories. Finally, a multivariate analysis was carried out with the multiple regression tests, using the stepwise method for academic achievement (GPA) and non-verbal intelligence as dependent variables (the emotions were not included separately in this analysis). The Cronbach’s alpha test was performed for all the subscales measured. All statistical analyses were carried out with the software SPSS v. 25.0 and a *p*-value ≤ 0.05 was considered as significant. 

## 3. Results 

### 3.1. Descriptive Results

A total of 175 students that failed the same subject twice attended in a student support service program. All of them were invited to participate, however, from these, 124 students (70.86%) accepted the invitation to participate and completed all the measurements. From these, 65 (52.41%) were men, the mean age ± SD (range) was: 23.12 ± 3.75 (18–45) years old, the majority of them (69.40%) had a job, did not have children (81.46%) and had a romantic partner (53.20%). According to the university study programs, they were studying 7 health sciences degrees: physical culture and sport (28%), nursing (21%), psychology (14.5%), medicine (14.5%), 4 different technician careers (11%), dentistry (7%) and nutrition (4%). The descriptive statistics of personal, psychological and academic variables are presented in Table 1.

### 3.2. Bivariate Correlations

In the comparison of GPA and non-verbal intelligence between sex (*t*-test), we did not find significant differences; likewise, no differences were found between the group of students with or without a romantic partner, neither for those who have versus who do not have children, neither for those who have a job versus who do not have it (*p* > 0.05); although for these 2 last variables, borderline differences were found in non-verbal intelligence, being higher for the students who do not have children compared with those who have them (85.43 ± 9.30 vs. 82.96 ± 6.37, *p* = 0.076), and for those students who have a job compared with those who do not have it (86.17 ± 9.16 vs. 82.24 ± 7.56, *p* = 0.092).

In the bivariate correlations, including both sexes, between all the variables included and GPA and non-verbal intelligence, and including the above referenced variables (codified as continuous variables), we observed that GPA presented low but significant positive correlations with non-verbal intelligence, preparatory GPA, the admission exam test, having a romantic partner and number of diseases. Additionally, non-verbal intelligence presented a positive moderate correlation with the admission exam test and low but significant positive correlations with preparatory GPA, GPA, age, having a job, schooling, maternal schooling, monthly extra money, somatization, openness and autonomy (Table 2). A low but significant negative correlation was observed between non-verbal intelligence and the number of siblings and agreeableness as a personality trait. In addition, non-verbal intelligence presented a tendency of a positive correlation with the number of diseases (r = 0.165, *p* = 0.07), and depression (r = 0.169, *p* = 0.06) and a tendency of a negative correlation with sleep satisfaction (r = −0.166, *p* = 0.06) and sleep quality (r = −0.156, *p* = 0.09). 

### 3.3. Correlations in Women 

When we correlated the variables by sex (Table 2), we observed that women presented a significant positive correlation between GPA and non-verbal intelligence (r = 0.535), admission exam test (r = 0.445) and preparatory GPA (r = 0.381), and also had tendencies of positive correlations with weekly physical activity hours (r = 0.209, *p* = 0.113), positive relations with others (r = 0.248, *p* = 0.06) and emotion perception (r = 0.224, *p* = 0.09). In addition, non-verbal intelligence presented a significant positive correlation with the admission exam test (r = 0.660), preparatory GPA (r = 0.366), maternal schooling (r = 0.430), monthly extra money (r = 0.328), number of diseases (r = 0.331), weekly physical activity hours (r = 0.271), somatization (r = 0.312), anxiety (r = 0.265), and positive tendencies with age (r = 0.229, *p* = 0.08), paternal schooling (r = 0.216, *p* = 0.10) academic stress (r = 0.218, *p* = 0.09), depression (r = 0.223, *p* = 0.09) and openness (r = 0.247, *p* = 0.06). A significant negative correlation was observed between non-verbal intelligence and agreeableness (r = −0.260). 

In the multivariate analysis for GPA, we found that non-verbal intelligence and preparatory GPA were the only significant associated variables included (Table 3) that showed a moderate R of the model = 0.624. The multivariate analysis for non-verbal intelligence in women included the admission exam test (β = 0.699), monthly extra money (β = 0.305) and smoking frequency (negatively) (β = −0.189) (data not shown in tables), with a R of the model = 0.754. 

### 3.4. Correlations in Men 

In the case of men, positive significant correlations were observed between GPA and having a romantic partner (r = 0.275), and positive tendencies were observed with having a job (r = 0.224, *p* = 0.08), emotion regulation (r = 0.201, *p* = 0.108), self-motivation (r = 0.189, *p* = 0.132) and neuroticism (r = 0.180, *p* = 0.151). In the case of non-verbal intelligence, positive significant correlations were found with the admission exam test (r = 0.546), having a job (r = 0.292) and autonomy (r = 0.261), and positive tendencies were found with daily study hours (r = 0.228, *p* = 0.06) and depression (r = 0.181, *p* = 0.149). A significant negative correlation was observed between non-verbal intelligence and agreeableness (r = −0.262) and a negative tendency with the number of siblings (r = −0.238, *p* = 0.06). 

In the multivariate analysis for GPA in men, we found that the most associated variables were the admission exam test, self-motivation, neuroticism, having a romantic partner, having children (negatively) and weekly physical activity hours (negatively), shown in Table 4, which showed a moderate multivariate r = 0.603. The multivariate analysis for non-verbal intelligence in men included the admission exam test (β = 0.589) and maternal schooling (β = 0.231), with a moderate multivariate r = 0.576, data not shown in tables. 

## 4. Discussion 

To the best of our knowledge, this is the first study that associates a wide number of variables, including psychological and academic ones, with the current GPA and non-verbal intelligence in university students and specifically, in students with low academic performance. In the descriptive values, we observed that the mean of the percentile of non-verbal intelligence was low (19.33), which in part explains the constant failing in this group of students. 

When we analyze the bivariate associations with GPA, we observed that women presented a higher correlation between non-verbal intelligence and GPA than men (r = 0.535 vs. r = 0.163), in whom, other variables, such as having a romantic partner (r = 0.275) or having a job (r = 0.224) were more correlated. This suggests that in men, non-verbal intelligence is not the highest predictor of GPA in university students and other factors are more relevant. In this regard, it is important to consider that, contrary to the expected, having a job was positively associated with GPA in both sexes, being higher in men than in women. As non-verbal intelligence, preparatory GPA and the admission exam test were also more correlated with GPA in women than in men. In general, these results coincide with other studies performed in children that showed that non-verbal intelligence has been the strongest associated variable to GPA [5,6]; however, no differences by sex were reported. 

Different to the expected, sleep satisfaction and sleep quality did not show a correlation or tendency with GPA; however, we observed that a previous report [6] did not show an association between sleep efficiency and GPA in children (r = 0.05), and another report only showed associations between GPA and morningness chronotype (r = 0.14) and an earlier midpoint of sleep (r = −0.22), but not with the average sleep length (r = 0.04) in children [5]. This suggests that sleep satisfaction or sleep quality are not associated with GPA in these populations (children and university), however, other variables associated to sleep, as previously mentioned, could be associated. 

On the other hand, although frequency of alcohol consumption did not show association or tendency with GPA in any sex, the smoking frequency showed a tendency of a very low correlation, but only in women (r = −0.158, *p* = 0.231). 

With respect to the psychological variables studied, we observed that for women, the emotional variables: emotion perception (r = 0.224, *p* = 0.09) and positive relation with others (r = 0.248, *p* = 0.06) presented positive tendencies in the correlation with GPA that were not observed in men, in whom, self-motivation (r = 0.189, *p* = 0.132) and emotion regulation (r = 0.201, *p* = 0.108) showed a positive tendency of correlation with GPA; self-motivation was also included in the multivariate analysis for GPA in men. These differences are important in order to perform intervention programs to increase GPA in this population, considering that different programs should be performed depending on the sex of the students. These results coincide with other reports that showed low but significant positive associations between emotional intelligence and learning strategies [27] and examinations [28] in health sciences university students; however, these reports did not perform analyses by sex. In addition, contrary to a previous report in children [5], we did not find an association between conscientiousness and GPA in any sex. 

In the multivariate models for GPA performed by sex, we observed that, in women, only 2 intellectual variables were included: non-verbal intelligence and preparatory GPA. In men, many variables were part of the model, including positive associations with the admission exam test, self-motivation, neuroticism, having a romantic partner and negative associations with having children and weekly physical activity hours, which confirms the influence of many variables in GPA (including personal, psychological and behavioral) in men compared with women. In this sense, intervention programs addressing emotion perception and positive relations with others, as well as healthy lifestyle habits may be more useful for women, and programs addressing self-motivation and emotion regulation could be more useful for men in order to increase GPA. However, longitudinal studies are needed in order to corroborate these possible causal associations. 

In the case of non-verbal intelligence, the correlation with having a job is related with the monthly extra money and is an expected association, considering that higher non-verbal intelligence increases the opportunity of getting a job and earning more money. Again, we observed that the correlation between non-verbal intelligence and monthly extra money was much higher (and only significant) in women than in men. 

In addition, as previously mentioned, we observed a positive correlation between physical activity hours and non-verbal intelligence only in women; in this regard, although aerobic fitness has shown to improve cognitive functions in young adults [29], another study showed that sedentary time predicted higher values of fluid non-verbal intelligence in children, while physical activity diminished them [30]. In addition, a third study showed that specific sedentary activities (watching television and driving) are related with lower scores of fluid non-verbal intelligence, but computer-use time is related with higher ones [31]. In this sense, future longitudinal studies that specify the type of physical and sedentary activities of the students are needed in order to relate them with the modification of non-verbal intelligence. 

As an unexpected finding, we observed a correlation between non-verbal intelligence and somatization (r = 0.194), which was higher and significant in women (r = 0.312), as well as a tendency with the number of diseases (r = 0.165, *p* = 0.07) that reached significance in women (r = 0.331). This could be related with the tendencies (not significant) to a negative correlation between non-verbal intelligence and sleep satisfaction (r = −0.166, *p* = 0.06) and sleep quality (r = −0.156, *p* = 0.09), and a tendency of a positive correlation with depression (r = 0.169, *p* = 0.06) which was higher in women (0.223, *p* = 0.09). This was considering that these variables had a correlation with somatization and the number of diseases: sleep satisfaction: r = −0.384, *p* < 0.01 and r = −0.212, *p* < 0.05; sleep quality: r = −0.563, *p* < 0.01 and r = −0.328, *p* < 0.01, and depression: r = 0.620, *p* < 0.01 and r = 0.452, *p* < 0.01, respectively (data not shown in tables). In addition, women had a positive significant correlation between non-verbal intelligence and anxiety (r = 0.265) and a positive tendency with academic stress (r = 0.218, *p* = 0.10) which was not found in men. These correlations also explain the higher correlations between non-verbal intelligence with somatization and the number of diseases in women than in men. 

These results coincide with a previous report showing a negative correlation between sleep duration on weekends and non-verbal intelligence scores in healthy children [32]. On the other hand, although a meta-analysis showed a negative correlation between cognitive functions and subsequent self-reported depression [33], another report found a higher number of psychological and physical diseases including depression and psychiatric and self-immune diseases in individuals with high intelligence when compared to the general population [34]. Interestingly, we found similar results but with the low intelligence population. In this case, more studies that differentiate men from women and use specific instruments to measure depression are needed. 

With respect to the psychological variables, according to the hypothesis, the positive correlations between non-verbal intelligence and the subscale of personality: openness (r = 0.210), mainly in women (r = 0.247), and a low negative correlation with the subscale of personality: agreeableness (r = −0.266), coincide with a previous report in the German adult population, where the authors found a positive correlation between fluid and crystallized non-verbal intelligence with openness (curiosity for fluid intelligence and aesthetic sensitivity for crystallized intelligence), and a negative correlation between crystallized intelligence and agreeableness [11]. 

The main limitation of this study was the lack of a control group with normal academic performance that could have permitted us to perform comparisons of the studied variables between groups and identify the differences in the variables related with academic performance; likewise, the inclusion of different study programs and stages on which the students were currently studying formed a more heterogeneous group that could have biased the results obtained. Nevertheless, the high number of variables, which included the analysis by sex and the poorly investigated population, permitted us to find new and potentially useful associations that can lead to further research and intervention programs in this population, in order to improve their academic achievement. 

## 5. Conclusions

In conclusion, we found that academic performance was mainly associated with non-verbal intelligence in women but not in men, in whom, other variables, including having a romantic partner and having a job, were more associated. Other sex differences were found: in women, the positive relations with others and emotion perception were marginally correlated with GPA; while in men, emotion regulation and self-motivation showed a tendency of correlation with GPA. In addition, in the multivariate analysis for men, many variables were included: admission exam test, self-motivation, neuroticism, having a romantic partner, having children (negatively) and weekly physical activity hours (negatively); while in women only 2 variables were included: non-verbal intelligence and preparatory GPA. Globally, these results emphasize the contribution of psychological variables to GPA in both genders, being of special interest, the social support provided by others or by a romantic partner; as well as the contribution of emotional abilities to GPA, including the emotion perception, self-motivation and emotion regulation. These results indicate that these associated variables can be increased in further studies in order to increase GPA.

## Figures and Tables

**Table 1:** Personal, psychological and academic/intellectual variables of the participants.

Variable	All Students (*n* = 124)	Women (*n* = 59)	Men (*n* = 65)	Possible Range
**Academic and intellectual variables**				
GPA, mean ± SD	84.46 ± 4.94	84.76 ± 4.94	84.19 ± 4.96	≤100
Preparatory GPA, mean ± SD	84.09 ± 6.99	84.44 ± 7.55	83.77 ± 6.47	≤100
Admission exam test, mean ± SD	61.14 ± 16.48	58.90 ± 16.37	63.17 ± 16.43	≤100
University Entrance Score (UES), mean ± SD	145.24 ± 20.02	143.34 ± 20.68	146.95 ± 19.39	≤200
Non-verbal intelligence (BETA-4 IQ), mean ± SD	84.97 ± 8.86	84.12 ± 8.55	85.74 ± 9.13	≤155
Non-verbal intelligence percentile, mean ± SD	19.34 ± 15.29	17.83 ± 14.57	20.71 ± 15.89	≤100
**Personal variables**				
Male sex, *n* (%)	65 (52.41)	-	-	-
Age, mean ± SD (range)	23.15 ± 3.75 (18–45)	22.25 ± 3.75	23.96 ± 3.57	-
With romantic partner, *n* (%)	66 (53.20)	29 (49.20)	37 (56.90)	-
With children, *n* (%)	23 (18.54)	11 (18.60)	12 (18.46)	-
With job, *n* (%)	86 (69.40)	38 (64.40)	48 (73.84)	-
Schooling, *n* (%)				-
- Secondary	6 (4.80)	4 (6.80)	2 (3.08)	
- Preparatory	77 (62.10)	39 (66.10)	38 (58.46)	
- University (Bachelor’s degree)	41 (33.10)	16 (27.10)	25 (38.46)	
Maternal Schooling, *n* (%)				-
- Elementary school	11 (8.87)	3 (5.10)	8 (12.30)	
- Secondary	38 (30.65)	18 (30.50)	20 (30.80)	
- Preparatory	31 (25.00)	14 (23.70)	17 (26.15)	
- University (Bachelor´s degree)	38 (30.65)	23 (39.00)	15 (23.07)	
- Master in Science	4 (3.22)	1 (1.70)	3 (4.61)	
- Ph.D. degree	2 (1.61)	0 (0.00)	2 (3.07)	
Paternal Schooling, *n* (%)				-
- Elementary school	16 (12.90)	8 (13.60)	8 (12.30)	
- Secondary	34 (27.42)	16 (27.10)	18 (27.70)	
- Preparatory	34 (27.42)	16 (27.10)	18 (27.70)	
- University (Bachelor´s degree)	29 (23.39)	15 (25.40)	14 (21.50)	
- Master in Science	10 (8.06)	3 (5.10)	7 (10.80)	
- Ph.D. degree	1 (0.81)	1 (1.70)	0 (0.00)	
Siblings, mean ± SD (range)	2.28 ± 1.39 (0–7)	2.39 ± 1.21	2.18 ± 1.54	-
Monthly extra money, mean ± SD	2.05 ± 0.60	1.94 ± 0.57	2.13 ± 0.61	0–5
Daily study hours, mean ± SD (range)	5.21 ± 3.11 (1–15)	5.40 ± 3.17	5.03 ± 3.07	-
Daily free hours, mean ± SD (range)	3.13 ± 2.05 (0–12)	3.12 ± 2.03	3.12 ± 2.08	-
Daily recreative hours, mean ± SD (range)	1.33 ± 1.02 (0–6)	1.14 ± 0.95	1.49 ± 1.06	-
Number of diseases, mean ± SD (range)	2.22 ± 2.04 (0–9)	3.17± 2.04	1.35 ± 1.61	0–17
**Behavioral variables**				
Weekly physical activity hours, mean ± SD (range)	4.70 ± 4.69 (0–28)	3.33 ± 3.38	1.35 ± 1.61	-
Frequency of drug consumption, mean ± SD	1.10 ± 0.26	1.09 ± 026	1.10 ± 0.26	1–6
Frequency of smoking, mean ± SD	2.19 ± 1.96	2.32 ± 1.99	2.07 ± 1.93	1–6
Frequency of alcoholism, mean ± SD	3.36 ± 1.99	3.18 ± 2.05	3.52 ± 1.94	1–6
Quality of food intake (Mini-ECCA), mean ± SD	6.31 ± 2.61	7.08 ± 2.69	5.61 ± 2.33	1–12
Self-reported BMI, mean ± SD (range)	25.15 ± 5.44 (15.06–40.82)	25.09 ± 5.38	25.21 ± 5.53	-
Sleep satisfaction (OVIEDO scale), mean ± SD	3.98 ± 1.40	3.83 ± 1.41	4.11 ± 1.38	1–7
Sleep quality (OVIEDO scale), mean ± SD	3.43 ± 1.04	3.30 ± 1.17	3.54 ± 0.90	1–5
**Psychological variables**				
Somatization (PHQ-15), mean ± SD	1.58 ± 0.36	1.71 ± 0.37	1.46 ± 0.30	1–3
Academic stress (SISCO), mean ± SD	2.34 ± 0.67	2.41 ± 0.71	2.28 ± 0.64	1–5
Depression (PHQ-9), mean ± SD	1.91 ± 0.69	2.10 ± 0.73	1.75 ± 0.60	1–4
Personality (NEO-FFI), mean ± SD				1–5
- Neuroticism	2.34 ± 0.87	2.56 ± 0.91	2.14 ± 0.77	
- Extraversion	3.34 ± 0.85	3.10 ± 0.75	3.54 ± 0.89	
- Agreeableness	3.51 ± 0.69	3.59 ± 0.73	3.44 ± 0.65	
- Openness	3.55 ± 0.77	3.59 ± 0.71	3.52 ± 0.82	
- Conscientiousness	3.63 ± 0.70	3.60 ± 0.76	3.67 ± 0.65	
Emotional intelligence (TEIQUE), mean ± SD				1–7
- Self-motivation	5.11 ± 1.16	5.00 ± 1.23	5.21 ± 1.08	
- Emotion regulation	4.45 ± 1.20	4.29 ± 1.16	4.59 ± 1.22	
- Emotion perception	4.86 ± 1.38	4.69 ± 1.42	5.02 ± 1.34	
Psychological well-being (PWB), mean ± SD				1–6
- Self-acceptance	4.34 ± 1.21	4.06 ± 1.30	4.59 ± 1.07	
- Positive relations with others	4.30 ± 1.14	4.14 ± 1.21	4.43 ± 1.06	
- Environmental mastery	4.20 ± 0.96	4.13 ± 0.98	4.27 ± 0.94	
- Personal growth	4.70 ± 0.97	4.72 ± 1.07	4.68 ± 0.87	
- Purpose in life	4.43 ± 1.21	4.32 ± 1.35	4.54 ± 1.06	
- Autonomy	4.24 ± 0.99	4.05 ± 1.06	4.41 ± 0.89	
Positive and negative emotions (PSS), mean ± SD				1–5
- Positive emotions	3.77 ± 0.63	3.64 ± 0.62	3.88 ± 0.62	
- Negative emotions	2.51 ± 0.70	2.70 ± 0.70	2.34 ± 0.66	
Anxiety (GAD-7), mean ± SD	2.02 ± 0.70	2.16 ± 0.72	1.90 ± 0.67	1–4
Optimism (LOT-R), mean ± SD	3.53 ± 0.69	3.50 ± 0.73	3.55 ± 0.65	1–5

GPA: Grade point average, IQ: intellectual quotient, BMI: body mass index. Monthly extra money was measured with 5 ordered categories from 1 = nothing to 5 = more than USD 150. Frequency of drug consumption, smoking and alcoholism were measured from 1 = never to 6 = many times in the week. Quality of food intake (Mini-ECCA scale) was measured from 1 = very low quality to 12 = very high quality; sleep satisfaction (OVIEDO scale) was measured from 1 = very unsatisfied to 7 = very satisfied; sleep quality (OVIEDO scale) was measured from 1 = very low quality to 5 = very high quality; somatization (PHQ-15) was measured from 1 = without disturbance to 3 = much disturbance; academic stress (SISCO) was the mean of the 8 academic stressors that were measured from 1 = never to 5 = always, depression (PHQ-9) was measured from 1 = no day to 4 = almost all the days; the 5 personality subscales (NEO-FFI) were measured from 1 = totally disagree to 5 = totally agree; the 3 subscales of emotional intelligence (TEIQUE) were measured from 1 = totally disagree to 7 = totally agree; the 6 subscales of PWB scale were measured from 1 = totally disagree to 6 = totally agree; positive and negative emotions (PSS) were measured from 1 = totally disagree to 6 = totally agree; anxiety (GAD-7) was measured from 1 = not at all to 4 = almost all the days; optimism (LOT-R) was measured from 1 = totally disagree to 5 = totally agree.

**Table 2:** Correlations of studied variables with non-verbal intelligence and GPA.

Variable	Including All the Students (*n* = 124)		Women (*n* = 59)		Men (*n* = 65)	
	Non-Verbal Intelligence	GPA	Non-Verbal Intelligence	GPA	Non-Verbal Intelligence	GPA
**Sex (Female = 1, Male = 2)**	0.102	−0.110	-	-	-	-
**Age**	0.274 **	0.107	0.229	0.138	0.277 **	0.168
**With romantic partner (No = 0, Yes = 1)**	0.091	0.220 *	0.211	0.167	−0.056	0.275 *
**With children (No = 0, Yes = 1)**	−0.108	−0.085	−0.150	−0.041	−0.091	−0.175
**Number of children (*n* = 23, women = 11, men = 12)**	−0.123	−0.084	−0.358	−0.162	0.034	0.130
**With job (No = 0, Yes = 1)**	0.220 *	0.166	0.148	0.135	0.292 *	0.224
**Schooling**	0.193 *	0.103	0.200	0.122	0.162	0.123
**Maternal Schooling**	0.237 **	−0.002	0.430 **	0.109	0.087	−0.130
**Paternal Schooling**	0.108	−0.031	0.216	0.059	−0.007	−0.014
**Siblings**	−0.205 *	−0.038	−0.129	0.054	−0.238	−0.160
**Monthly extra money**	0.200 *	−0.022	0.328 *	−0.039	0.055	0.019
**Daily study hours**	0.138	0.080	0.048	−0.015	0.228	0.158
**Daily free hours**	−0.036	−0.109	0.037	−0.068	−0.128	−0.136
**Daily recreative hours**	−0.011	−0.035	0.070	−0.002	−0.126	−0.035
**Number of diseases**	0.165	0.195 *	0.331 *	0.166	0.094	0.168
**Weekly physical activity hours**	0.046	0.029	0.271 *	0.209	−0.071	−0.198
**Frequency of drug consumption**	−0.058	−0.010	0.051	−0.015	0.062	0.028
**Frequency of smoking**	0.041	−0.122	−0.014	−0.158	0.112	−0.088
**Frequency of alcoholism**	−0.032	−0.028	−0.009	−0.081	−0.053	0.019
**Quality of food intake**	0.024	0.032	0.173	0.122	−0.068	−0.146
**Self-reported BMI**	0.071	0.085	0.158	0.027	0.000	0.140
**Sleep satisfaction**	−0.166	−0.045	−0.214	−0.080	−0.140	0.000
**Sleep quality**	−0.156	0.005	−0.158	0.011	−0.135	−0.034
**Somatization (PHQ−15)**	0.194 *	0.164	0.312 *	0.186	0.164	0.108
**Academic stress (SISCO)**	0.123	0.041	0.218	−0.041	0.045	0.108
**Depression (PHQ−9)**	0.169	0.040	0.223	−0.036	0.181	0.105
**Neuroticism**	0.089	0.093	0.142	−0.008	0.091	0.180
**Extraversion**	−0.079	0.041	−0.192	0.105	−0.051	0.018
**Agreeableness**	−0.266 **	−0.052	−0.260 *	−0.048	−0.262 *	−0.076
**Openness**	0.210 *	0.077	0.247	0.156	0.120	−0.064
**Conscientiousness**	−0.085	0.060	−0.086	0.113	−0.067	0.045
**Self-motivation**	0.007	0.127	0.018	0.079	−0.019	0.189
**Emotion regulation**	0.171	0.127	0.182	0.059	0.144	0.201
**Emotion perception**	0.099	0.065	0.123	0.224	0.096	−0.129
**Self-acceptance**	−0.076	0.021	−0.102	0.124	−0.090	−0.057
**Positive relations with others**	0.133	0.159	0.172	0.248	0.069	0.078
**Environmental mastery**	−0.027	−0.040	−0.085	−0.012	0.004	−0.063
**Personal growth**	0.055	0.044	0.062	0.068	0.048	0.009
**Purpose in life**	−0.115	−0.011	−0.085	0.029	−0.167	0.043
**Autonomy**	0.183 *	0.059	0.086	0.108	0.261 *	0.038
**Positive emotions**	0.038	0.146	0.011	0.193	0.032	0.132
**Negative emotions**	0.048	−0.032	0.172	−0.093	−0.014	−0.007
**Anxiety (GAD−7)**	0.112	−0.004	0.265 *	0.023	0.021	−0.047
**Optimism (LOT-R)**	0.087	0.109	0.001	0.197	0.161	0.024
**GPA**	0.326 **	-	0.535 **	-	0.163	-
**Non-verbal intelligence**	-	0.326 **	-	0.535 **	-	0.163
**Preparatory GPA**	0.241 **	0.191 *	0.366 **	0.381 **	0.131	−0.013
**Admission exam test**	0.602 **	0.323 **	0.660 **	0.445 **	0.546 **	0.233

* *p*-value < 0.05 and ** <0.01. *p*-values calculated with Spearman and Pearson correlation tests.

**Table 3:** Multiple regression analysis for GPA in women.

Variable	Beta	Beta Coefficient	*p*-Value	Change in R^2^
Constant	45.33	-	0.000	-
Non-verbal intelligence	0.279	0.463	0.000	0.326
Preparatory GPA	0.186	0.273	0.014	0.063

GPA: Grade point average. R of the model: 0.624.

**Table 4:** Multiple regression analysis for GPA in men.

Variable	Beta	Beta Coefficient	*p*-Value	Change in R^2^
Constant	64.94	-	0.000	-
Admission exam test	0.083	0.277	0.011	0.087
Self-motivation	1.846	0.399	0.001	0.055
Neuroticism	1.939	0.294	0.016	0.079
With romantic partner	3.543	0.348	0.003	0.048
With children	−3.343	−0.254	0.025	0.049
Physical activity	−0.227	−0.225	0.040	0.047

GPA: Grade point average. R of the model: 0.603.

## Data Availability

Data that support the findings of the study are available from the corresponding author upon reasonable request.

## Supplementary Materials

The following supporting information can be downloaded at: https://www.mdpi.com/article/10.3390/ijerph19084804/s1, Supplementary File S1: items included for emotional intelligence subscales measurement (TIEQUE scale).
