# Supplementary material for: Association of Health and Psychological Factors with Academic Achievement and Non-Verbal Intelligence in University Students with Low Academic Performance: The Influence of Sex
Source: Int J Environ Res Public Health. 2022 Apr 15;19(8):4804. doi: 10.3390/ijerph19084804 (PMC9029569; doi:10.3390/ijerph19084804)
Supplement: Supplementary file 1 [file ijerph-19-04804-s001.zip › ijerph-1643256-supplementary.pdf]

Items included in the emotional intelligence subscales of the TEIQUE scale

Self-motivation: items 35, 73, 77, 129 and 142

Emotion regulation: items 36, 84, 90, 100, 117 and 140

Emotion perception: items 9, 32, 37, 45 and 113

Assertiveness: items 34, 64, 111, 115, 144 and 151

The items were selected from the Spanish version of TEIQUE:
https://www.psychometriclab.com/adminsdata/files/TEIQue_v.1.5_Spanish_versión%20final_COLOR.pdf
